# Development of a Prokaryotic Universal Primer for Simultaneous Analysis of *Bacteria* and *Archaea* Using Next-Generation Sequencing

**DOI:** 10.1371/journal.pone.0105592

**Published:** 2014-08-21

**Authors:** Shunsuke Takahashi, Junko Tomita, Kaori Nishioka, Takayoshi Hisada, Miyuki Nishijima

**Affiliations:** TechnoSuruga Laboratory Co., Ltd., Shizuoka-shi, Shizuoka, Japan; International Atomic Energy Agency, Austria

## Abstract

For the analysis of microbial community structure based on 16S rDNA sequence diversity, sensitive and robust PCR amplification of 16S rDNA is a critical step. To obtain accurate microbial composition data, PCR amplification must be free of bias; however, amplifying all 16S rDNA species with equal efficiency from a sample containing a large variety of microorganisms remains challenging. Here, we designed a universal primer based on the V3-V4 hypervariable region of prokaryotic 16S rDNA for the simultaneous detection of *Bacteria* and *Archaea* in fecal samples from crossbred pigs (Landrace×Large white×Duroc) using an Illumina MiSeq next-generation sequencer. *In-silico* analysis showed that the newly designed universal prokaryotic primers matched approximately 98.0% of *Bacteria* and 94.6% of *Archaea* rRNA gene sequences in the Ribosomal Database Project database. For each sequencing reaction performed with the prokaryotic universal primer, an average of 69,330 (±20,482) reads were obtained, of which archaeal rRNA genes comprised approximately 1.2% to 3.2% of all prokaryotic reads. In addition, the detection frequency of *Bacteria* belonging to the phylum *Verrucomicrobia*, including members of the classes *Verrucomicrobiae* and *Opitutae*, was higher in the NGS analysis using the prokaryotic universal primer than that performed with the bacterial universal primer. Importantly, this new prokaryotic universal primer set had markedly lower bias than that of most previously designed universal primers. Our findings demonstrate that the prokaryotic universal primer set designed in the present study will permit the simultaneous detection of *Bacteria* and *Archaea*, and will therefore allow for a more comprehensive understanding of microbial community structures in environmental samples.

## Introduction

The analysis of 16S rRNA (16S rDNA) provides valuable phylogenetic information for the comparison of microbial diversity in environmental samples. A number of molecular biological techniques based on 16S rDNA gene sequence diversity have been developed for investigating microbial community structure. Among these techniques, fingerprinting methods, such as denaturing gradient gel electrophoresis (DGGE) [1]–[3] and terminal-restriction fragment length polymorphism (T-RFLP) [4]–[6], are frequently used due to their ease of use and relatively low cost [7]. The construction of 16S rDNA clone libraries is also extensively used to study microbial community structure [2], [4], [8]. However, as these culture-independent approaches are estimated to detect only 0.1%∼1% of the total microbial population, they are not optimal for the analysis of subdominant microbial species or groups [9], [10].

In recent years, the development of next-generation sequencing (NGS) technologies has permitted in-depth sequencing and data analyses of various types of environmental samples [11]–[15] at a deeper level than possible with standard molecular biological techniques [16]. In particular, Illumina-based strategies, which provide paired reads of the same DNA fragment, offer multiplexing capability and generate large amounts of sequence data [16], [17]. Of the commercially available Illumina platforms, the MiSeq sequencer has the greatest potential for 16S rDNA sequence studies, because it generates sequence reads of up to 600 bp and has a performance to cost ratio that is manageable for average-sized research laboratories [18]. The generation of longer sequence reads is an important feature of this platform, as they are easier to assign to taxonomic groups [19]. In addition, because the MiSeq Illumina platform allows for deeper sequencing than conventional approaches, it allows for the detection and analysis of subdominant microbial species or groups [11]. Therefore, the combination of this NGS technology with robust prokaryotic universal primer sets will allow for the more accurate determination of the *Bacteria* to *Archaea* ratio than conventional approaches. Several studies have successfully applied prokaryotic universal primer sets and NGS platforms for microbial community structure analysis of environmental and clinical samples, including Artic sea ice, soil, and urinary catheters [20]–[22]. However, the phylogenetic specificity and degree of bias of these prokaryotic universal primers remain unclear.

In the present study, we designed universal primer sets for the specific detection of the V3-V4 regions of prokaryotic 16S rDNA, including the domains *Bacteria* and *Archaea*. In addition, we developed a simultaneous analysis system for *Bacteria* and *Archaea* based on Illumina MiSeq next-generation sequencing technology.

## Materials and Methods

### Fecal samples

Fecal samples were collected from three 11-month-old crossbred pigs (Landrace×Large white×Duroc), which were housed on a farm maintained by Kyoto Prefectural University. The breeding of pigs adhered to the Guidelines Concerning the Care and Use of Laboratory Animals of the Animal Experiment Committee of Kyoto Prefectural University. Because the feces were collected non-invasively, a permit number was not required for the research. Feces samples were kindly collected by Dr. K. Ushida and Dr. S. Tsuchida (Kyoto Prefectural University) and were then transported to our laboratory. The feces from individual pigs were divided into 5-g samples and then frozen at −80°C until use.

### DNA extraction

Frozen fecal samples were thawed on ice, 100 mg of each sample was suspended in 4 M guanidium thiocyanate, 100 mM Tris-HCl (pH 9.0), and 40 mM EDTA, and the samples were then beaten with zirconia beads using a FastPrep FP100A instrument (MP Biomedicals, USA). DNA was extracted from the bead-treated suspensions using a Magtration System 12GC and GC series MagDEA DNA 200 (Precision System Science, Japan). DNA concentrations were estimated by spectrophotometry using an ND-1000 instrument (NanDrop Technologies, USA), and the final concentration of the DNA sample was adjusted to 10 ng/µL.

### Development of a prokaryotic universal primer set

Previously published universal PCR primer sets targeting the V3-V4 region of bacterial and archaeal rDNA were modified to increase the detection rate of prokaryotes [23], [24]. Multiple alignments of 16S rDNA sequences obtained from the DDBJ/GenBank/EMBL database for selected reference organisms were performed using the Clustal W program with default settings [25]. Based on the alignment results, primer sequences that gave the fewest mismatches in the V3-V4 hypervariable region of the 16S rRNA gene were selected as prokaryotic universal primers. The coverage of the previous and present primer sets was checked against good quality sequences (as defined based on Pintail scores by the RDP database) of greater than 1,200 bp and ≤1 mismatches [26] in the RDP database (Release 10) using the feature Probe Match program [27]. The primer sequences and associated PCR assays used in this study are listed in Table 1.

**Table 1:** Primers and thermal cycling profiles used in this study.

Target	Primer name	Oligonucleotide sequence (5'–3')	Experiment	Reference
Prokaryote 16S rRNA	Pro341F	AATGATACGGCGACCACCGAGATCTACACTCTTTCCCTACACGACGCTCTTCCGATCTCCTACGGGAGGCAGCAG **CCTACGGGNBGCASCAG**	PCR for NGS	Modified Takai [23]
	Pro805R	CAAGCAGAAGACGGCATACGAGATNNNNNNGTGACTGGAGTTCAGACGTGTGCTCTTCCGATCT **GACTACNVGGGTATCTAATCC**		Modified Herlemann [24]
Bacteria 16S rRNA	341F	AATGATACGGCGACCACCGAGATCTACACTCTTTCCCTACACGACGCTCTTCCGATCTCCTACGGGAGGCAGCAG **CCTACGGGAGGCAGCAG**	PCR for NGS	Muyzer [44]
	R806	CAAGCAGAAGACGGCATACGAGATNNNNNNGTGACTGGAGTTCAGACGTGTGCTCTTCCGATCT **GGACTACHVGGGTWTCTAAT**		Caporaso [45]
	341F	CCTACGGGAGGCAGCAG	q-PCR	Muyzer [44]
	R806	GGACTACHVGGGTWTCTAAT		Caporaso [45]
	8F	AGAGTTTGATCCTGGCTCAG	PCR	Edwards [46]
	1510R	GGTTACCTTGTTACGACTT		Reysenbach [47]
Archaea 16S rRNA	ARC344F	AATGATACGGCGACCACCGAGATCTACACTCTTTCCCTACACGACGCTCTTCCGATCTCCTACGGGAGGCAGCAG **ACGGGGYGCAGCAGGCGCGA**	PCR for NGS	Raskin [48]
	Arch806R	CAAGCAGAAGACGGCATACGAGATNNNNNNGTGACTGGAGTTCAGACGTGTGCTCTTCCGATCT **GGACTACVSGGGTATCTAAT**		Takai [23]
	ARC344F	ACGGGGYGCAGCAGGCGCGA	q-PCR	Raskin [48]
	Arch806R	GGACTACVSGGGTATCTAAT		Takai [23]
	21F	TTCCGGTTGATCCYGCCGGA	PCR	DeLong [49]
	1510R	GGTTACCTTGTTACGACTT		Reysenbach [47]
Illumina adapters (sequencing)	Miseq_F	AATGATACGGCGACCACCGAGAT	qPCR	This study
	Miseq_R	CAAGCAGAAGACGGCATACGAGAT		
*Methanobacteria*	Pig Methano F	TCCGCAATGTGAGAAATCGC	qPCR	This study
	Pig Methano R	TACCCTGGGAGTACCTCTAACCTCT		
*Thermoplasmata*	Pig Thermo F	GAGGGAATTCCTAGTGCTAGGACA	qPCR	This study
	Pig Thermo R	ATCAAACCGGCTACGAACGTT		

Underlined regions indicate Illumina adapter sequences. Bold-face font indicates PCR primer region, which is preceded by a linker sequence. Poly-N string in forward primer denotes barcode sequence.

Barcode sequences used in this study (5′-3′): GATCTG, TCAAGT, CTGATC, AAGCTA, GTAGCC, TACAAG, CGTGAT, ACATCG, and GCCTAA.

### Illumina library generation

The V3-V4 region of 16S rDNA was amplified using Pro341F/Pro805R for *Prokaryotes*, 341F/R806 for *Bacteria*, and ARC344F/Arch806R for *Archaea* primer sets (Table 1). In addition to the V3-V4 specific priming regions, these primers were complementary to standard Illumina forward and reverse primers. The reverse primer also contained a 6-bp indexing sequence (Table 1) to allow for multiplexing. Amplification primers were designed with Illumina adapters.

To reduce the formation of spurious by-products during the amplification process, the touchdown PCR method for thermal cycling was used with a Rotor-Gene Q quantitative thermal cycler (Qiagen, Germany) [28]. The reaction mixture (25 µL) contained 10 ng genomic DNA, MightyAmp for Real Time (SYBR Plus) (Takara, Japan), and 0.25 µM of each primer. The PCR reaction conditions for amplification of DNA were as follows: initial denaturation at 98°C for 2 min, followed by 35 cycles of annealing beginning at 65°C and ending at 55°C for 15 sec, and extension at 68°C for 30 sec. The annealing temperature was lowered 1°C every cycle until reaching 55°C, which was used for the remaining cycles. PCR products were purified through a MultiScreen PCR_u96_ filter plate (Merck Millipore, USA) and analyzed using a Bioanalyzer DNA 1000 Chip Kit (Agilent Technologies, USA) to detect primer-dimers and determine the average molecular weight of each product. The purified products were quantified by real-time quantitative PCR (q-PCR) on a Rotor-Gene Q quantitative thermal cycler using MightyAmp for Real Time (SYBR Plus), 0.2 µM of each primer, which were derived from Illumina adapters (Table 1), and serially diluted PhiX control library (Illumina, USA) as a standard. The PCR reaction conditions for quantification of each PCR product were as following: initial denaturation at 98°C for 2 min, followed by 30 cycles of denaturation at 98°C for 10 sec, annealing at 60°C for 15 sec, and extension at 68°C for 30 sec. The quantification step was used to determine the concentration of the amplified libraries and to confirm the presence of suitable primers for Illumina sequencing.

### Illumina sequencing and quality filtering

Each multiplexed library pool was spiked with 25% phiX control to improve base calling during sequencing, as recommended by Illumina for the pooling of two libraries. Sequencing was conducted using a paired-end, 2×250-bp cycle run on an Illumina MiSeq sequencing system and MiSeq Reagent Nano Kit version 2 (500 Cycle) chemistry. After sequencing was complete, image analysis, base calling, and error estimation were performed using Illumina Real-Time Analysis software (version 1.17.28).

Paired-end sequencing with read lengths of 251 bp was performed. After demultiplexing, a clear overlap in the paired-end reads was observed. This allowed paired reads to be joined together with the fastq-join program (http://code.google.com/p/ea-utils/). Only reads that that had quality value (QV) scores of ≥20 for more than 99% of the sequence were extracted for further analysis. All sequences with ambiguous base calls were discarded. The nucleotide sequence dataset was deposited in the Sequence Read Archive of the DNA Data Bank of Japan (DDBJ) under the accession number DRA002295.

### 16S rDNA-based taxonomic analysis

Analyses of sequence reads were performed manually using the Ribosomal Database Project (RDP) Multiclassifier tool [19], which is available from the RDP website (http://rdp.cme.msu.edu/classifier/). Reads obtained in the FASTA format were assigned to class levels with an 80% confidence threshold.

### Real-time quantitative PCR

Quantitation of all *Bacteria*, *Archaea*, class *Thermoplasmata*, and class *Methanobacteria* in the pig intestinal tract was performed using real-time quantitative PCR (qPCR). To obtain standards for the qPCR, purified genomic DNA from *Escherichia coli* JCM 1649^T^ and *Methanosarcina acetivorans* DSM 2834^T^ was amplified using primer pairs 8F and 1510R or 21F and 1510R, respectively (Table 1). Extracted fecal DNA was amplified using primer pairs Pig Thermo F and Pig Thermo R or Pig Methano F and Pig Methano R (Table 1). The PCR reaction mixture (25 µL) contained 20 ng genomic DNA, 2×MightyAmp Buffer Ver.2 (Takara), 0.25 µM of each primer, and 1.25 units of MightyAmp DNA Polymerase (Takara). The cycling conditions were as follows: initial denaturation at 98°C for 2 min, followed by 35 cycles of 98°C for 10 s, 55°C for 15 s, and 68°C for 1 min. The amplification products (3 µL) were mixed with 1 µL EZ-Vision One DNA Dye (Amresco Inc., USA) and then separated by electrophoresis on 2% agarose gels to confirm the production of a single product of the expected molecular weight. The PCR products were purified using Econo Spin IIa (Gene Design, Japan) and then cloned into pGEM-T Easy vector (Promega, USA) for the transformation of *E. coli* HST08 Premium Competent Cells (Takara). Positive transformants were selected on LB agar supplemented with ampicillin (100 µg/ml), and a single colony that was verified by PCR to contain a plasmid with the expected insert DNA was grown in 5 ml LB medium supplemented with ampicillin (100 µg/ml) overnight. The culture was centrifuged at 7,610×*g* to pellet the cells, and plasmid DNA was then extracted from cells using a QIAprep Spin miniprep kit according to the manufacturer's instructions (Qiagen). The purified plasmid was quantified using a ND-1000 instrument. The number of 16S rDNA copies present in the plasmid preparation was estimated based on the DNA concentration and molecular mass of pGEM-T Easy with the target insert, as previously described [29]. For the calculation, the expected weight in Daltons (g/mol) of the plasmid construct was first determined using the equation: (length of the double-stranded product in base pairs [bp]) * (330 Da×2 nucleotides/bp). The obtained value was then divided by Avogadro's number (6.022×10^23^) to give the number of grams per molecule, which was then divided by the total amount of the plasmid preparation to give the total number of copies present in the sample. The purified plasmid DNA solution was serially diluted 10-fold to give solutions ranging from 10^3^ to 10^7^ copies/µL. The serially diluted samples were used to generate a standard curve that was used to estimate the copy number of the target group for each qPCR reaction.

Q-PCR was performed on a Rotor-Gene Q quantitative thermal cycler using SYBR *Premix Ex Taq* II (Tli RNaseH Plus) (Takara). Each reaction mixture (20 µL) contained 20 ng extracted DNA and 0.2 µM of each primer. The cycling conditions were as follows: initial denaturation at 95°C for 30 sec, followed by 35 cycles of 95°C for 5 sec, 60°C (all *Bacteria* and *Archaea*), 58°C (*Thermoplasmata*) or 55°C (*Methanobacteria*) for 20 sec, and 72°C for 20 sec. For each reaction, the positive control and negative water control were assayed together with the samples. The melting curves of the amplified DNA were generated to verify the specificity of the reaction. The ratio of the total archaeal population to that of prokaryotes was estimated from total bacterial and archaeal copy numbers using the following equation:
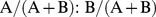
(1)


where A is the total copy number of *Bacteria* and B is the total copy number of *Archaea*. Similarly, the ratio of *Thermoplasmata* to *Methanobacteria* was also calculated using equation (1), with A representing the total copy number of *Thermoplasmata* and B representing the total copy number of *Methanobacteria*.

## Results

### Development and coverage of the prokaryotic universal primer

We modified the universal primers Pro341F and Pro805R, which target the V3–V4 hypervariable region of 16S rDNA of both *Bacteria* and *Archaea*, to improve the coverage of existing sequences in the RDP database. The results of *in-silico* analysis for primer specificity against 16S rDNA sequences of the newly designed primer, and the domain *Bacteria*- and *Archaea*-specific universal primers are presented in Table 2. The new prokaryotic universal primer matched approximately 98.0% of *Bacteria* and 94.6% of *Archaea* rRNA gene sequences present in the RDP database (release 10). The match percentages of the previously reported *Bacteria* (341F/R806) and *Archaea* (ARC344F/Arch806R) domain-specific primers were 97.4% and 63.4%, respectively. Thus, the coverage of *Archaea* sequences by the newly designed prokaryotic universal primer was markedly higher than that of the archaeal specific primer.

**Table 2:** *In-silico* analysis for the coverage rate of predicted primer sets.

Primer set	Target	Hits/queries (≤1 mismatch)	Coverage rate*
Pro341F/Pro805R	*Bacteria*	1230518/1255860	98.0%
	*Archaea*	22318/23588	94.6%
341F/R806	*Bacteria*	1223304/1255860	97.4%
ARC344F/Arch806R	*Archaea*	14966/23588	63.4%
Uni340F/Uni806R [23]	*Bacteria*	1224925/1255860	97.5%
	*Archaea*	22319/23588	94.6%
PRK341F/PRK806R [40]	*Bacteria*	1223340/1255860	97.4%
	*Archaea*	22132/23588	93.8%

Primers used in the present and previous studies were submitted to the ‘Probe Match program' facility of the Ribosomal Database Project (http://www.rdp.cme.msu.edu/) to evaluate specificity.

*The coverage of each primer set was calculated from the total number of matching sequences.

### NGS analysis using the prokaryotic universal primer

**Figure 1:**
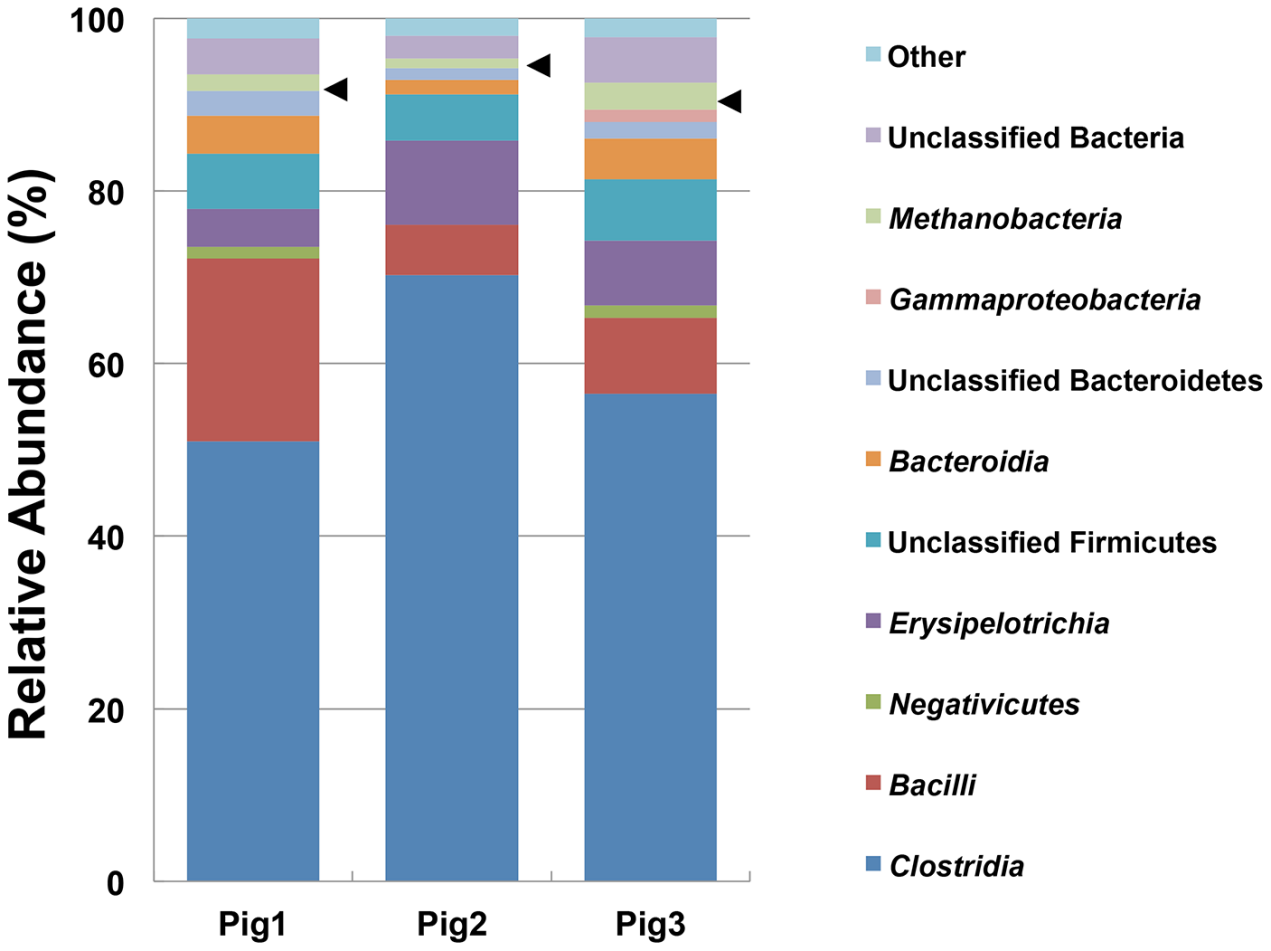
Class-level NGS analysis results for microbial diversity in pig fecal samples using the prokaryotic universal primer. The bar charts show the taxonomic profiles obtained for each of the three pig fecal samples. The arrowheads indicate archaeal reads (*Methanobacteria*).

To determine whether *Bacteria* and *Archaea* could be simultaneously detected using the newly designed prokaryotic universal primer, NGS analysis was performed for pig fecal samples. Using this primer and MiSeq platform combination, an average of 69,330 (±20,482) reads were obtained for each sequencing reaction. The reads were then analyzed at the class level using the RDP Multiclassifier tool (Fig. 1). The NGS analysis indicated that members of the class *Clostridia* were the most dominant taxonomic group in all samples. As shown in Fig. 1, reads corresponding to archaeal rRNA genes comprised approximately 1.2% to 3.2% of prokaryotic reads for all samples analyzed using the prokaryotic universal primer.

### Comparison of the prokaryotic abundance ratio determined by NGS and q-PCR

**Figure 2:**
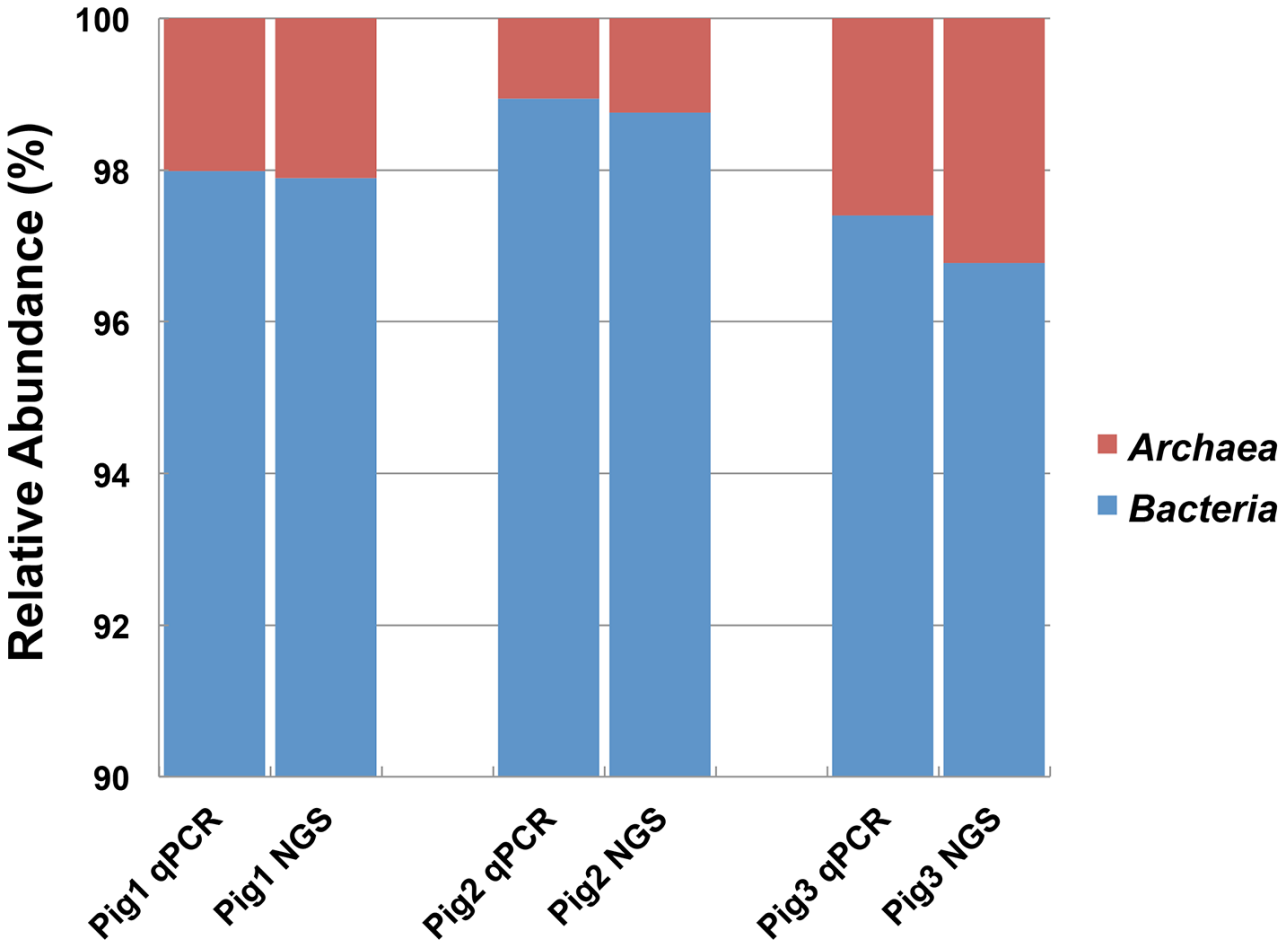
Relative abundance ratios of *Bacteria* and *Archaea* estimated by NGS with the prokaryotic universal primer. The results of NGS analysis for the three pig fecal samples are compared to those obtained by real-time quantitative PCR (qPCR).

We next analyzed whether the detection rate of *Archaea* was influenced by primer bias by calculating the relative abundance of *Bacteria* versus *Archaea* sequences using q-PCR. The abundance ratio of *Archaea* was approximately 1.1% to 2.6% of all prokaryotes, and the abundance ratios of *Bacteria* and *Archaea* were consistent with the q-PCR results (Fig. 2). From this result, it was determined that the prokaryotic universal primer did not display strong primer bias and was able to accurately reflect the relative abundance of *Bacteria* and *Archaea*.

### PCR bias of the prokaryotic universal primer for the domain *Bacteria*

**Figure 3:**
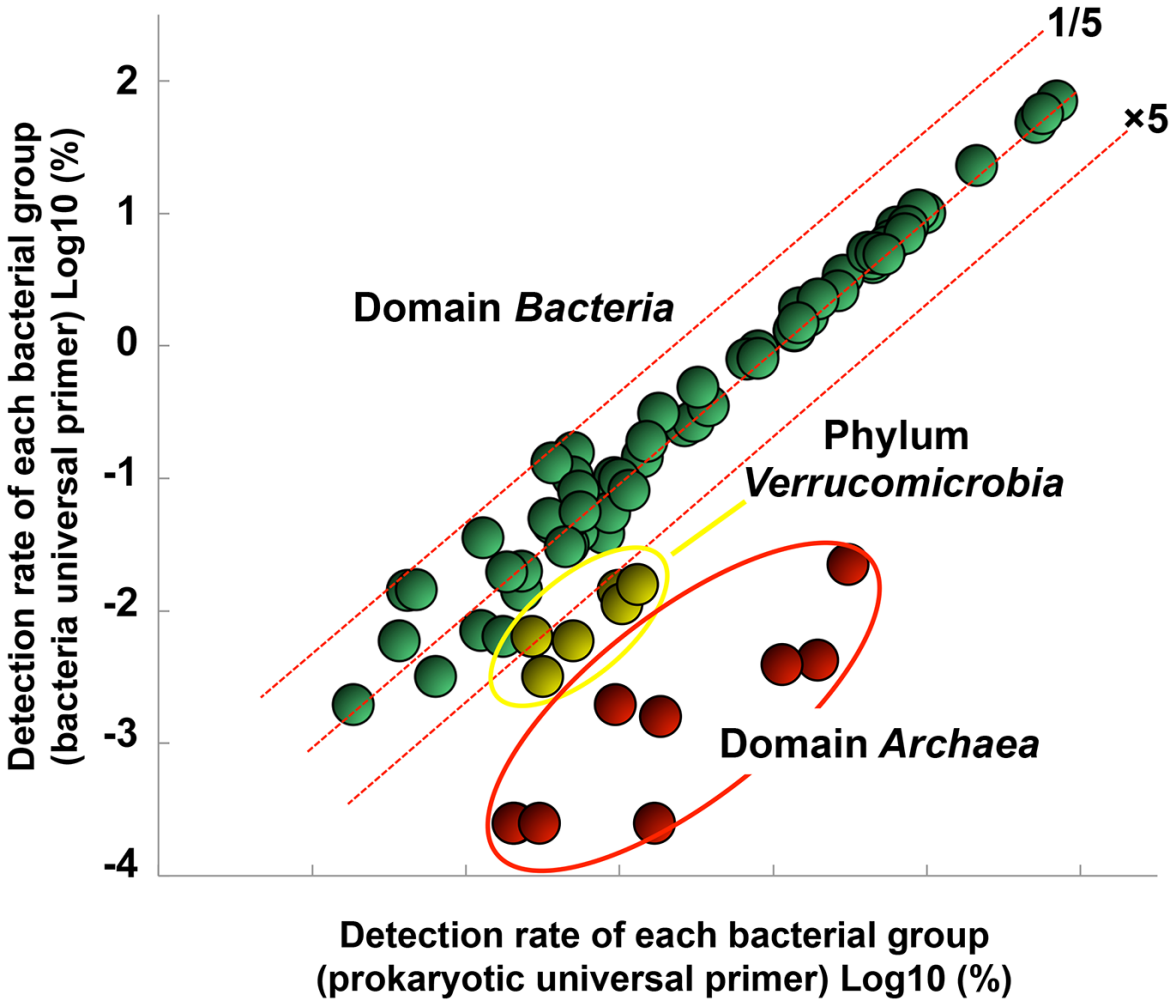
PCR bias of the prokaryotic universal primer for members of the domain *Bacteria* (Class-level NGS analysis).

**Figure 4:**
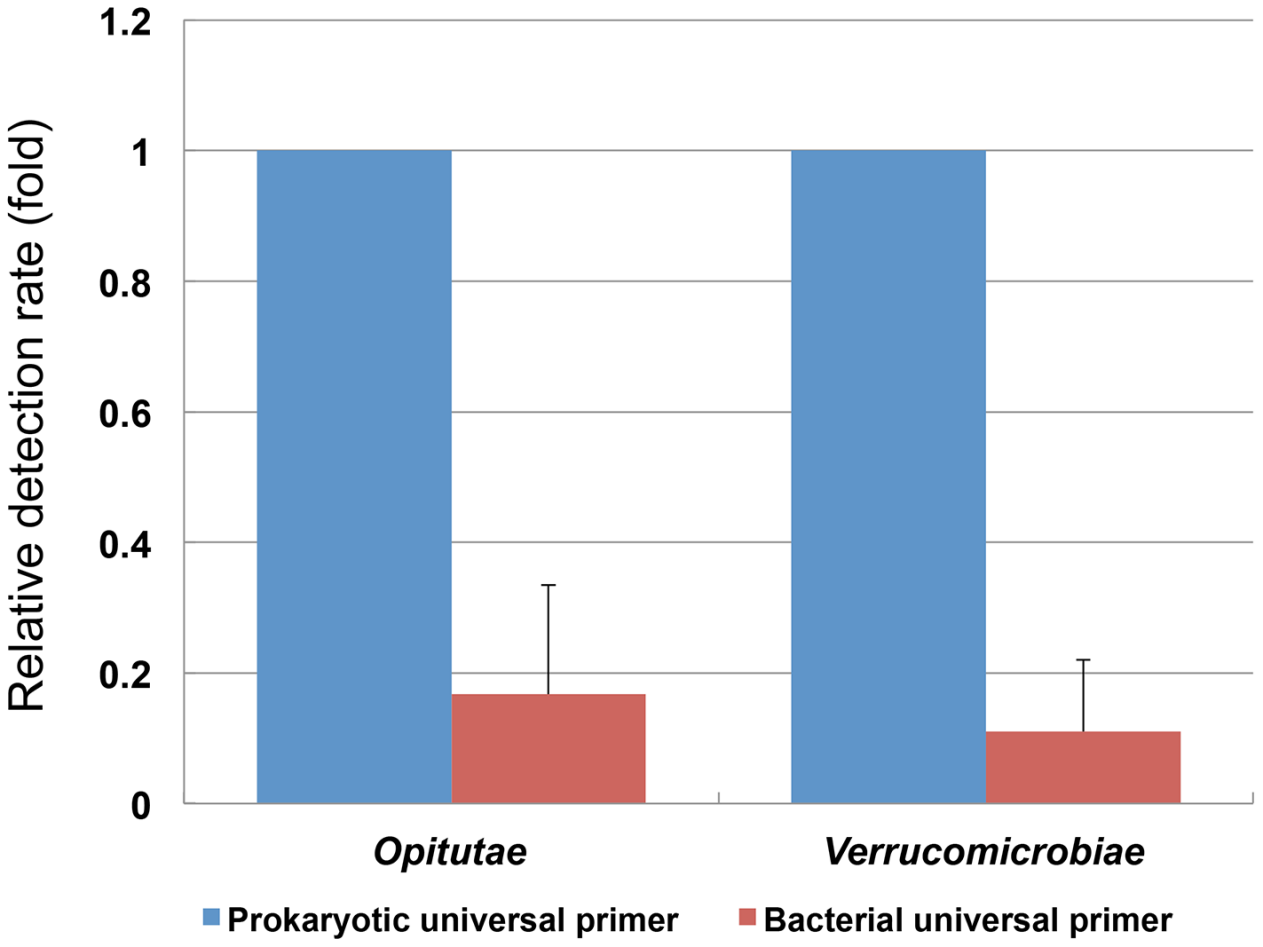
Relative abundance ratios of members of the classes *Verrucomicrobiae* and *Opitutae* for NGS analyses using the prokaryotic and bacterial universal primers.

To evaluate the effect of primer bias on the abundance estimation of bacterial taxonomic groups, the NGS analysis results obtained using the bacterial and newly developed prokaryotic universal primers were compared. The estimated abundance ratios of the bacterial taxonomic groups based on the sequences obtained with either the prokaryotic or bacterial universal primer were in agreement for nearly all of the dominant bacterial taxonomic groups (abundance-ratio >0.001%) (Fig. 3). However, archaeal sequences were also detected in the analyses performed with the bacterial universal primer, indicating that primer bias existed, although the detection frequency of *Archaea* was reduced compared to that obtained with the prokaryotic universal primer. In addition, the detection frequency of *Bacteria* belonging to the phylum *Verrucomicrobia*, including members of the classes *Verrucomicrobiae* and *Opitutae*, was higher in the NGS analysis using the prokaryotic universal primer than that performed with the bacterial universal primer (Fig. 4).

### PCR bias of the prokaryotic universal primer for the domain *Archaea* by NGS analysis and q-PCR

**Figure 5:**
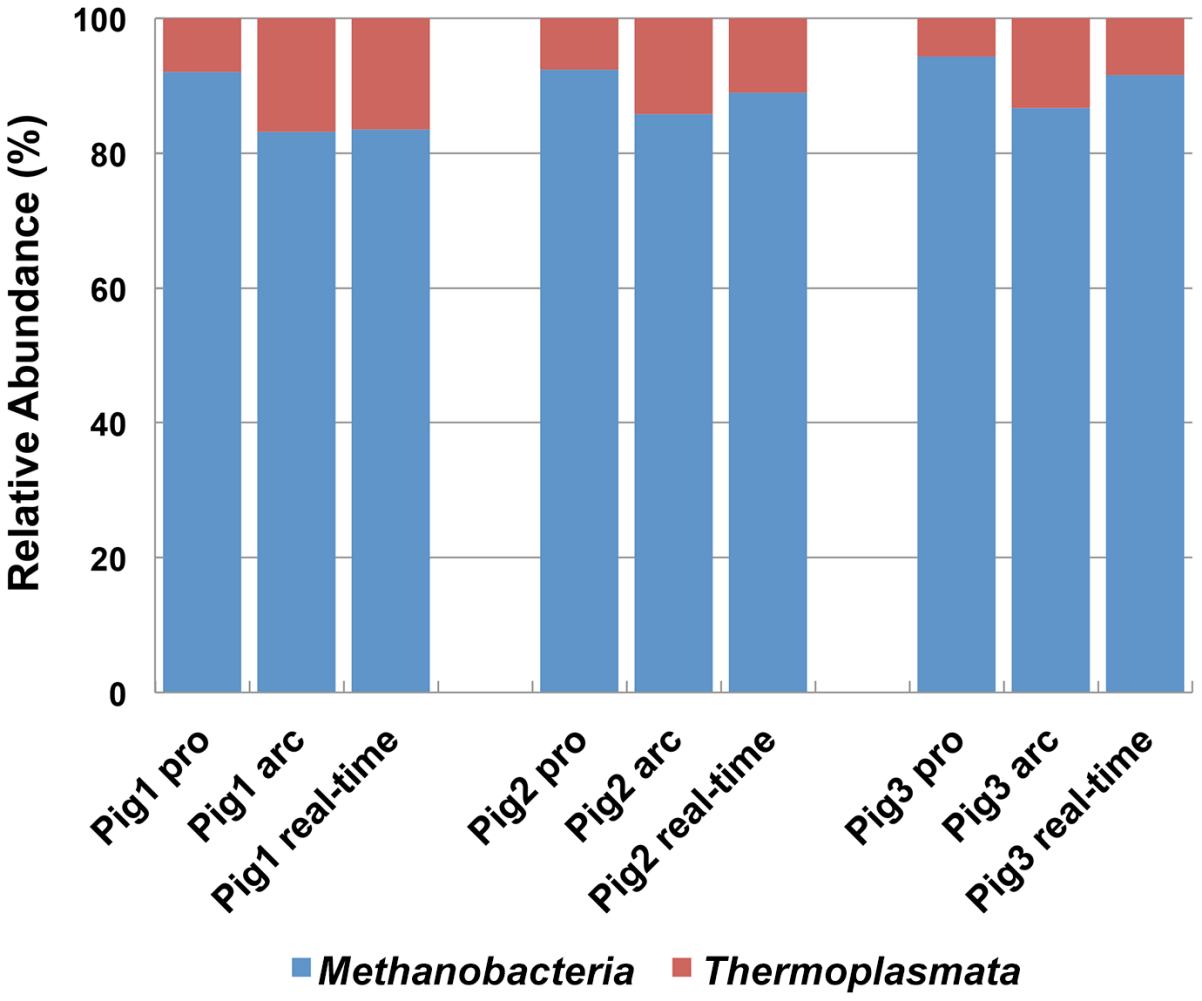
Relative abundance ratios of the classes *Thermoplasmata and Methanobacteria* estimated by NGS with the prokaryotic universal primer (pro) and archaeal universal primer (arc).

To evaluate the effect of primer bias on the abundance estimation of the two major archaeal taxonomic groups (classes *Thermoplasmata* and *Methanobacteria*) detected in the NGS analysis, the results obtained using the archaeal and prokaryotic universal primers were compared. The detection frequency of members of the class *Methanobacteria* was higher in the NGS analysis using the prokaryotic universal primer than that in the analysis performed with the archaeal universal primer (Fig. 5).

Class-specific primer sets for q-PCR analysis were also designed to estimate and compare the relative abundance ratios of the classes *Thermoplasmata* and *Methanobacteria* with the NGS analysis. The abundance ratios of these two classes in *Archaea* were similar for the NGS analysis using the prokaryotic primer and the q-PCR analysis using the class-specific primer sets (Fig. 5), although the relative abundance of *Methanobacteria* was markedly higher using the prokaryotic universal primer in the first fecal samples (Fig. 5, Pig1). In contrast, the estimated abundance ratios of the classes *Thermoplasmata* and *Methanobacteria* by q-PCR markedly differed when compared to the NGS analysis performed with the archaeal universal primer. Specifically, the ratio of *Methanobacteria* in the analysis with the archaeal universal primer was lower than that estimated using class-specific primers in the q-PCR analysis.

## Discussion

In the present study, we designed and examined the utility of a prokaryotic universal primer for the simultaneous amplification of bacterial and archaeal 16S rDNA using an Illumina MiSeq next-generation sequencer. We found that the newly developed primer set matches 98.0% of *Bacteria* and 94.6% of *Archaea* rRNA gene sequences in the RDP database and allows for the simultaneous analysis of *Bacteria* and *Archaea* in pig fecal samples. In addition, the prokaryotic universal primer has less bias than previously described universal primers, a characteristic that is critical for obtaining accurate microbial composition data. Thus, the newly designed prokaryotic universal primer set is expected to permit the simultaneous detection of *Bacteria* and *Archaea* in environmental samples, and will therefore improve the cost and efficiency of microbial community structure analysis using NGS technology.

In the NGS analyses with the newly designed prokaryotic universal primer, all identified taxonomic groups were also detected in the sequencing analysis with the bacterial universal primer; however, the detection frequency of several taxonomic groups markedly differed between the two primer sets. Notably, bacteria belonging to the classes *Verrucomicrobiae* and *Opitutae* were detected in the fecal samples at a nearly five-fold higher frequency with the prokaryotic universal primer. The prokaryotic universal primer was designed based on a universal primer (Uni340F, 5′-CCTACGGGRBGCASCAG-3′) described by Takai and Horikoshi [23]. However, we speculated that members of *Verrucomicrobiae* and *Opitutae*, which are increasingly being recognized as abundant species in various environments, were difficult to detect using primer 341F due to a single base mismatch with the 16S rRNA gene at the 9th base from the 5' end of the bacterial 341F primer. Thus, we modified primer Uni340F to remove the single mismatch (5′-CCTACGGG**N**BGCASCAG′) and confirmed that the relative detection rate of *Verrucomicrobiae* and *Opitutae* with the modified prokaryotic universal primer increased five-fold compared to the bacterial universal primer. Several studies have also demonstrated that even a single internal nucleotide mismatch in the V3-V4 target region of the primer can drastically alter PCR bias [_ENREF_1430-32]. For example, a comparison of two primer sets targeting the V3-V4 region of the 16S rRNA gene showed that vastly different fractions of the bacterial community in activated sludge were detected [31], [33]. In addition, differences in the nucleotide sequence of the primer and the V3 region strongly affected the microbial community structure [14]. We also found that the prokaryotic universal primer set gave different abundance ratios for the classes *Thermoplasmata* and *Methanobacteria* compared to the archaeal universal primer in the NGS analysis of the three pig fecal samples. The results of q-PCR with specific primers for these two classes suggested that the prokaryotic universal primer more accurately estimated the abundance ratios than the archaeal universal primer. To confirm these findings, however, the analysis of more environmental samples using both primers sets is needed.

NGS analysis with the prokaryotic universal primer indicated that members of the class *Clostridia* were the most dominant taxonomic group in all fecal samples. This finding is consistent with a previous analysis of pig intestinal microflora based on 16S rDNA that found *Clostridiales* was the dominant bacterial group [34]–[36]. In addition, we found that archaeal rRNA genes comprised approximately 1.2% to 3.2% of all prokaryotic reads in the analysis with the prokaryotic universal primer. Increasing evidence suggests that archaea are widely distributed in the environment [37], [38], and it is well-known that archaea are part of the intestinal microbiota in humans [38], although a comprehensive analysis of the archaeal community structure has yet to be performed. However, to our knowledge, this is the first report of the relative abundance ratio of *Archaea* in the pig intestinal tract using an NGS approach. Several studies have examined microbial diversity in pigs using DGGE and 16S rDNA clone libraries [36], [39]. For example, a relatively recent study examined the microbial composition of aerosols in a swine confinement facility and found that all detected *Archaea* sequences belonged to methanogenic archaea, which is consistent with our present finding of abundant *Methanobacteria* 16S rRNA in pig fecal samples [39].

For the accurate determination of microbial community structure based on 16S rDNA sequence diversity, sensitive, robust, and unbiased PCR amplification of 16S rDNA is critical. In particular, a high match percentage of primers is critical to avoid bias in cultivation-independent investigations of microbial community structure. The present *in-silico* analysis revealed that the coverage rates of the newly designed universal prokaryotic primer for sequences in the RDP database were approximately 0.5% higher for *Bacteria* and 0.8% higher for *Archaea* than those of previously described universal primers [23], [40]. We also demonstrated that the universal prokaryotic primer set designed here has markedly lower bias than that of most previously applied universal primers [23], [40]. It was previously suggested that the presence of a single internal primer-template mismatch in the primer extension sequence can result in a 1,000-fold underestimation of gene copy number, leading to an underrepresentation of the taxonomic groups that do not match the universal primer sequence [30]. As described above regarding the increased detection rate of *Verrucomicrobiae* and *Opitutae*, our universal prokaryotic primer set perfectly matched a higher number of 16S rRNA gene sequences in the examined fecal samples than the bacterial universal primer and therefore displayed less bias (Table 2). However, it also possible that the primer bias was due to inherent properties of the individual 16S rRNA sequences in the examined samples, including the G+C content, copy number, and melting temperature [41]–[43], and features affected by the amplification conditions, such as secondary structure and duplex stability. Thus, further optimization of the PCR conditions, such as amplification cycle number, primer and genomic DNA concentrations, and the type and amount of polymerase, are expected to further reduce PCR bias.

In conclusion, we have developed a universal prokaryotic primer set that allows for the simultaneous analysis of *Bacteria* and *Archaea* with limited PCR bias. The match percentage of the newly designed universal prokaryotic primer for sequences in the RDP is higher than that of previously described universal primers, and the NGS analysis results for the pig fecal samples indicate that this primer set has increased sensitivity for members of the classes *Verrucomicrobiae* and *Opitutae*. Thus, when applied for NGS technology, the prokaryotic universal primer set designed here is expected to provide a more comprehensive understanding of microbial community structure in environmental samples with reduced time and cost.

## References

[1] Pereira e Silva MC, Dias AC, van Elsas JD, Salles JF (2012) Spatial and temporal variation of archaeal, bacterial and fungal communities in agricultural soils. PLoS One 7: e51554. 23284712 10.1371/journal.pone.0051554 PMC3527478

[2] Tang C, Madigan MT, Lanoil B (2013) Bacterial and archaeal diversity in sediments of west Lake Bonney, McMurdo Dry Valleys, Antarctica. Appl Environ Microbiol 79: 1034–1038. 23183970 10.1128/AEM.02336-12 PMC3568539

[3] Patra AK, Yu Z (2012) Effects of essential oils on methane production and fermentation by, and abundance and diversity of, rumen microbial populations. Appl Environ Microbiol 78: 4271–4280. 22492451 10.1128/AEM.00309-12 PMC3370521

[4] Zumsteg A, Luster J, Goransson H, Smittenberg RH, Brunner I, et al (2012) Bacterial, archaeal and fungal succession in the forefield of a receding glacier. Microb Ecol 63: 552–564. 22159526 10.1007/s00248-011-9991-8

[5] Winter C, Matthews B, Suttle CA (2013) Effects of environmental variation and spatial distance on *Bacteria*, *Archaea* and viruses in sub-polar and arctic waters. ISME J 7: 1507–1518. 23552622 10.1038/ismej.2013.56 PMC3721122

[6] Braker G, Ayala-del-Rio HL, Devol AH, Fesefeldt A, Tiedje JM (2001) Community structure of denitrifiers, *Bacteria*, and *Archaea* along redox gradients in Pacific Northwest marine sediments by terminal restriction fragment length polymorphism analysis of amplified nitrite reductase (*nirS*) and 16S rRNA genes. Appl Environ Microbiol 67: 1893–1901. 11282647 10.1128/AEM.67.4.1893-1901.2001 PMC92811

[7] Zoetendal EG, Collier CT, Koike S, Mackie RI, Gaskins HR (2004) Molecular ecological analysis of the gastrointestinal microbiota: a review. J Nutr 134: 465–472. 14747690 10.1093/jn/134.2.465

[8] Han M, Liu F, Zhang F, Li Z, Lin H (2012) Bacterial and Archaeal symbionts in the South China Sea sponge *Phakellia fusca*: community structure, relative abundance, and ammonia-oxidizing populations. Mar Biotechnol 14: 701–713. 22310803 10.1007/s10126-012-9436-5

[9] Nakayama J, Tanaka S, Songjinda P, Tateyama A, Tsubouchi M, et al (2007) Analysis of Infant intestinal microbiota by various kinds of molecular approaches: toward large scale epidemiological investigations correlating allergy development with intestinal microbiota. J Intestinal Microbiol 21: 129–142.

[10] Curtis TP, Head IM, Lunn M, Woodcock S, Schloss PD, et al (2006) What is the extent of prokaryotic diversity? Philos Trans R Soc Lond B Biol Sci 361: 2023–2037. 17028084 10.1098/rstb.2006.1921 PMC1764926

[11] Caporaso JG, Paszkiewicz K, Field D, Knight R, Gilbert JA (2012) The Western English Channel contains a persistent microbial seed bank. ISME J 6: 1089–1093. 22071345 10.1038/ismej.2011.162 PMC3358019

[12] Fouts DE, Szpakowski S, Purushe J, Torralba M, Waterman RC, et al (2012) Next generation sequencing to define prokaryotic and fungal diversity in the bovine rumen. PLoS One 7: e48289. 23144861 10.1371/journal.pone.0048289 PMC3492333

[13] Kiyohara M, Koyanagi T, Matsui H, Yamamoto K, Take H, et al (2012) Changes in microbiota population during fermentation of *narezushi* as revealed by pyrosequencing analysis. Bioscience Biotechnology and Biochemistry 76: 48–52. 10.1271/bbb.110424 22232244

[14] Pinto AJ, Raskin L (2012) PCR biases distort bacterial and archaeal community structure in pyrosequencing datasets. PLoS One 7: e43093. 22905208 10.1371/journal.pone.0043093 PMC3419673

[15] Yergeau E, Lawrence JR, Sanschagrin S, Waiser MJ, Korber DR, et al (2012) Next-generation sequencing of microbial communities in the Athabasca River and its tributaries in relation to oil sands mining activities. Appl Environ Microbiol 78: 7626–7637. 22923391 10.1128/AEM.02036-12 PMC3485728

[16] Shokralla S, Spall JL, Gibson JF, Hajibabaei M (2012) Next-generation sequencing technologies for environmental DNA research. Mol Ecol 21: 1794–1805. 22486820 10.1111/j.1365-294X.2012.05538.x

[17] Quail MA, Smith M, Coupland P, Otto TD, Harris SR, et al (2012) A tale of three next generation sequencing platforms: comparison of Ion Torrent, Pacific Biosciences and Illumina MiSeq sequencers. BMC Genomics 13: 341. 22827831 10.1186/1471-2164-13-341 PMC3431227

[18] Kozich JJ, Westcott SL, Baxter NT, Highlander SK, Schloss PD (2013) Development of a dual-index sequencing strategy and curation pipeline for analyzing amplicon sequence data on the MiSeq Illumina sequencing platform. Appl Environ Microbiol 79: 5112–5120. 23793624 10.1128/AEM.01043-13 PMC3753973

[19] Wang Q, Garrity GM, Tiedje JM, Cole JR (2007) Naive Bayesian classifier for rapid assignment of rRNA sequences into the new bacterial taxonomy. Appl Environ Microbiol 73: 5261–5267. 17586664 10.1128/AEM.00062-07 PMC1950982

[20] Bowman JS, Rasmussen S, Blom N, Deming JW, Rysgaard S, et al (2011) Microbial community structure of Arctic multiyear sea ice and surface seawater by 454 sequencing of the 16S RNA gene. ISME J 6: 11–20. 21716307 10.1038/ismej.2011.76 PMC3246233

[21] Eilers KG, Debenport S, Anderson S, Fierer N (2012) Digging deeper to find unique microbial communities: the strong effect of depth on the structure of bacterial and archaeal communities in soil. Soil Biology and Biochemistry 50: 58–65.

[22] Xu Y, Moser C, Al-Soud WA, Sorensen S, Hoiby N, et al (2012) Culture-dependent and -independent investigations of microbial diversity on urinary catheters. J Clin Microbiol 50: 3901–3908. 23015674 10.1128/JCM.01237-12 PMC3502987

[23] Takai K, Horikoshi K (2000) Rapid detection and quantification of members of the archaeal community by quantitative PCR using fluorogenic probes. Appl Environ Microbiol 66: 5066–5072. 11055964 10.1128/aem.66.11.5066-5072.2000 PMC92420

[24] Herlemann DP, Labrenz M, Jurgens K, Bertilsson S, Waniek JJ, et al (2011) Transitions in bacterial communities along the 2000 km salinity gradient of the Baltic Sea. ISME J 5: 1571–1579. 21472016 10.1038/ismej.2011.41 PMC3176514

[25] Thompson JD, Higgins DG, Gibson TJ (1994) CLUSTAL W: improving the sensitivity of progressive multiple sequence alignment through sequence weighting, position-specific gap penalties and weight matrix choice. Nucleic Acids Res 22: 4673–4680. 7984417 10.1093/nar/22.22.4673 PMC308517

[26] Nakayama J (2010) Pyrosequence-based 16S rRNA profiling of gastro-intestinal microbiota. Bioscience Microflora 29: 83–96.

[27] Maidak BL, Cole JR, Lilburn TG, Parker CT Jr, Saxman PR, et al (2001) The RDP-II (Ribosomal Database Project). Nucleic Acids Res 29: 173–174. 11125082 10.1093/nar/29.1.173 PMC29785

[28] Don RH, Cox PT, Wainwright BJ, Baker K, Mattick JS (1991) 'Touchdown' PCR to circumvent spurious priming during gene amplification. Nucleic Acids Res 19: 4008. 1861999 10.1093/nar/19.14.4008 PMC328507

[29] Whelan JA, Russell NB, Whelan MA (2003) A method for the absolute quantification of cDNA using real-time PCR. J Immunol Methods 278: 261–269. 12957413 10.1016/s0022-1759(03)00223-0

[30] Bru D, Martin-Laurent F, Philippot L (2008) Quantification of the detrimental effect of a single primer-template mismatch by real-time PCR using the 16S rRNA gene as an example. Appl Environ Microbiol 74: 1660–1663. 18192413 10.1128/AEM.02403-07 PMC2258636

[31] Klindworth A, Pruesse E, Schweer T, Peplies J, Quast C, et al (2013) Evaluation of general 16S ribosomal RNA gene PCR primers for classical and next-generation sequencing-based diversity studies. Nucleic Acids Res 41: e1. 22933715 10.1093/nar/gks808 PMC3592464

[32] Wu JH, Hong PY, Liu WT (2009) Quantitative effects of position and type of single mismatch on single base primer extension. J Microbiol Methods 77: 267–275. 19285527 10.1016/j.mimet.2009.03.001

[33] Fredriksson NJ, Hermansson M, Wilén B-M (2013) The choice of PCR primers has great impact on assessments of bacterial community diversity and dynamics in a wastewater treatment plant. PLoS ONE 8: e76431. 24098498 10.1371/journal.pone.0076431 PMC3788133

[34] Leser TD, Amenuvor JZ, Jensen TK, Lindecrona RH, Boye M, et al (2002) Culture-independent analysis of gut bacteria: the pig gastrointestinal tract microbiota revisited. Appl Environ Microbiol 68: 673–690. 11823207 10.1128/AEM.68.2.673-690.2002 PMC126712

[35] Snell-Castro R, Godon JJ, Delgenès JP, Dabert P (2005) Characterisation of the microbial diversity in a pig manure storage pit using small subunit rDNA sequence analysis. FEMS Microbiol Ecol 52: 229–242. 16329909 10.1016/j.femsec.2004.11.016

[36] Liu F, Wang S, Zhang J, Zhang J, Yan X, et al (2009) The structure of the bacterial and archaeal community in a biogas digester as revealed by denaturing gradient gel electrophoresis and 16S rDNA sequencing analysis. J Appl Microbiol 106: 952–966. 19187154 10.1111/j.1365-2672.2008.04064.x

[37] Wrede C, Dreier A, Kokoschka S, Hoppert M (2012) Archaea in symbioses. Archaea 2012: 596846. 23326206 10.1155/2012/596846 PMC3544247

[38] Bäckhed F, Ley RE, Sonnenburg JL, Peterson DA, Gordon JI (2005) Host-bacterial mutualism in the human intestine. Science 307: 1915–1920. 15790844 10.1126/science.1104816

[39] Nehmé B, Gilbert Y, Létourneau V, Forster RJ, Veillette M, et al (2009) Culture-independent characterization of archaeal biodiversity in swine confinement building bioaerosols. Appl Environ Microbiol 75: 5445–5450. 19561186 10.1128/AEM.00726-09 PMC2737903

[40] Yu Y, Lee C, Kim J, Hwang S (2005) Group-specific primer and probe sets to detect methanogenic communities using quantitative real-time polymerase chain reaction. Biotechnol Bioeng 89: 670–679. 15696537 10.1002/bit.20347

[41] Farrelly V, Rainey FA, Stackebrandt E (1995) Effect of genome size and *rrn* gene copy number on PCR amplification of 16S rRNA genes from a mixture of bacterial species. Appl Environ Microbiol 61: 2798–2801. 7618894 10.1128/aem.61.7.2798-2801.1995 PMC167554

[42] Fogel G, Collins C, Li J, Brunk C (1999) Prokaryotic genome size and SSU rDNA copy number: estimation of microbial relative abundance from a mixed population. Microbial Ecology 38: 93–113. 10441703 10.1007/s002489900162

[43] Ishii K, Fukui M (2001) Optimization of annealing temperature to reduce bias caused by a primer mismatch in multitemplate PCR. Appl Environ Microbiol 67: 3753–3755. 11472961 10.1128/AEM.67.8.3753-3755.2001 PMC93085

[44] Muyzer G, de Waal EC, Uitterlinden AG (1993) Profiling of complex microbial populations by denaturing gradient gel electrophoresis analysis of polymerase chain reaction-amplified genes coding for 16S rRNA. Appl Environ Microbiol 59: 695–700. 7683183 10.1128/aem.59.3.695-700.1993 PMC202176

[45] Caporaso JG, Lauber CL, Walters WA, Berg-Lyons D, Lozupone CA, et al (2011) Global patterns of 16S rRNA diversity at a depth of millions of sequences per sample. Proc Natl Acad Sci U S A 108 Suppl 14516–4522. 20534432 10.1073/pnas.1000080107 PMC3063599

[46] Edwards U, Rogall T, Blocker H, Emde M, Bottger EC (1989) Isolation and direct complete nucleotide determination of entire genes. Characterization of a gene coding for 16S ribosomal RNA. Nucleic Acids Res 17: 7843–7853. 2798131 10.1093/nar/17.19.7843 PMC334891

[47] Reysenbach AL, Wickham GS, Pace NR (1994) Phylogenetic analysis of the hyperthermophilic pink filament community in Octopus Spring, Yellowstone National Park. Appl Environ Microbiol 60: 2113–2119. 7518219 10.1128/aem.60.6.2113-2119.1994 PMC201609

[48] Raskin L, Stromley JM, Rittmann BE, Stahl DA (1994) Group-specific 16S rRNA hybridization probes to describe natural communities of methanogens. Appl Environ Microbiol 60: 1232–1240. 7517128 10.1128/aem.60.4.1232-1240.1994 PMC201464

[49] DeLong EF (1992) Archaea in coastal marine environments. Proc Natl Acad Sci U S A 89: 5685–5689. 1608980 10.1073/pnas.89.12.5685 PMC49357

